# Arabic exercise assessment and screening for you tool: development, validation, and cultural adaptation among older adults in Saudi Arabia

**DOI:** 10.3389/fmed.2026.1815705

**Published:** 2026-04-13

**Authors:** Saad M. Alsaad, Nasser M. AbuDujain, Norah Alrashoud, Nada Bin Obaid, Osama Abdulqader, Antoni Krupa, Barbara Resnick

**Affiliations:** 1 Department of Family and Community Medicine, College of Medicine, King Saud University, Riyadh, Saudi Arabia; 2 Prince Faisal Bin Bandar Chair for Geriatric Research, College of Medicine, King Saud University, Riyadh, Saudi Arabia; 3 College of Medicine, King Saud University, Riyadh, Saudi Arabia; 4 Tawuniya Insurance Company (Headquarters), Riyadh, Saudi Arabia; 5 Oxford Medical School, Oxford University, Oxford, United Kingdom; 6 School of Nursing, University of Maryland, Baltimore, MD, United States

**Keywords:** Arabic, EASY scale, older adults, physical activity, Rasch analysis, validation

## Abstract

**Background and aim:**

Regular physical activity is a key determinant of healthy aging; however, physical inactivity remains highly prevalent among older adults. Most existing screening tools emphasize risk assessment rather than providing individualized guidance for safe exercise. The Exercise Assessment and Screening for You (EASY) tool is an algorithm-based instrument designed to guide older adults toward appropriate physical activities based on their health conditions. In this study, we aimed to translate and culturally adapt the EASY tool into Arabic and evaluate its psychometric properties among older adults.

**Methods:**

We conducted an observational cross-sectional study at King Saud University Medical City, Riyadh (2025–2026), including adults aged ≥50 years. Participants completed an electronic survey that included demographic information, the Arabic EASY (Ar-EASY), the International Physical Activity Questionnaire–Short Form (IPAQ-SF), and the Physical Activity Scale for the Elderly (PASE). Participants were recruited via convenience sampling from family medicine and geriatric clinics, and analytic subsamples varied across psychometric analyses due to data availability.

**Results:**

A total of 127 older adults were included (median age: 60 years), of whom 63.8% were physically active. The Rasch analysis demonstrated generally acceptable item functioning and scale-targeting abilities. Confirmatory factor analysis supported the five-item model with excellent fit indices (CFI = 1.00, TLI = 1.08, RMSEA = 0.00). EASY scores were negatively correlated with IPAQ-SF scores (*r* = −0.31, *p* = 0.033) but not with PASE scores. The test–retest reliability was moderate (ICC = 0.58).

**Conclusion:**

The Ar-EASY scale showed acceptable psychometric properties and provided a brief, culturally adapted screening tool to support individualized physical activity guidance in older adults. Future studies should confirm its performance using larger, population-based samples.

## 1 Introduction

Regular physical activity is widely recognized as an important component of healthy aging. Multiple studies have demonstrated that even low to moderate levels of physical activity can lead to significant health benefits in older adults, including improved cardiovascular health, enhanced mobility, better cognitive function, and increased overall quality of life (1). However, physical activity remains low among older individuals, with rates declining with advancing age (2). One of the main factors contributing to low rates of physical activity among older adults is the belief that poor health and medical conditions are barriers to engaging in physical activity (3).

In Saudi Arabia, the rate of physical inactivity is high, particularly among older adults. A total of 66.6% of the Saudi population is physically inactive, and the majority belong to the 55–64 age group (4). Additionally, the WHO estimates that the population of Saudi Arabia aged 65 years and older will continue to increase and will comprise 18.4% of the total population by 2050 (5). This highlights the importance of prioritizing strategies to promote physical activity among older adults while considering their health conditions.

Various tools have been used to measure physical activity levels (6). Most of these tools focus on highlighting the potential risks and harms of physical activity without tailoring them to an individual's health status.

The Exercise Assessment and Screening for You (EASY) tool was developed by Resnick et al. (7) to support older adults, as well as their physicians and fitness professionals, in identifying the kinds of exercises or physical activities that would best suit them. This ensures that the type of activity chosen is suited to the person's current health condition, illness, or disability. The tool includes six screening questions that can be answered by older adults independently or with the assistance of a primary physician or fitness instructor. Additionally, this tool is algorithm-based and uses questionnaire responses to provide appropriate advice and guidance regarding physical activity. In contrast to other tools that highlight the risks of physical activity in older adults, the EASY tool focuses on the benefits of staying physically active and encourages older adults to maintain physical activity while considering their health conditions (7). The EASY has been translated into Spanish, Mandarin, and Hindi (8–10).

To the best of our knowledge, this tool has not yet been translated into or validated in Arabic. Therefore, in this study, we aimed to translate, culturally adapt, and assess the validity and reliability of the tool for Arabic-speaking populations. Existing physical activity instruments, such as the IPAQ and PASE, primarily quantify activity levels but do not provide individualized guidance regarding exercise safety or suitability in the presence of comorbidities. In contrast, the EASY tool is specifically designed as a screening and decision-support instrument that links health status to tailored physical activity recommendations. This distinction is particularly relevant in older adults, where clinical considerations often influence exercise participation. Therefore, the availability of a culturally adapted Arabic version of the EASY tool may help address an important gap in clinical practice by facilitating safe, personalized exercise guidance.

## 2 Methodology

### 2.1 Study design and participant recruitment

This observational, cross-sectional validation study was conducted at King Saud University Medical City in Riyadh, Saudi Arabia, between January 2025 and October 2025. The study included adults aged ≥50 years who attended family medicine and specialized geriatric clinics. Participants whose responses were provided by caregivers, those with communication barriers that precluded independent questionnaire completion, and those with cognitive impairments or memory loss were excluded.

Based on established methodological recommendations for scale validation, a sample size of 10–20 participants per item was considered adequate (11), using non-probability convenience sampling. Given that the EASY tool comprises 6 items, a minimum sample size of 60–120 participants was required. The evaluation of measurement properties was guided by the COSMIN (COnsensus-based Standards for the selection of health Measurement INstruments) framework for evaluating measurement properties.

Data collection was conducted by trained research assistants using a standardized administration procedure to ensure consistency across participants. Eligible participants were identified from the registry of staff and staff dependents and contacted electronically with an invitation to participate. Because recruitment occurred via a registry-based electronic distribution process, the exact number of individuals who viewed the invitation could not be determined; therefore, a precise response rate was unavailable.

### 2.2 Study questionnaire and variables

Participants completed an electronic survey consisting of three sections. The first section collected demographic and functional information, including age, sex, educational level, nationality, marital status, monthly income, functional mobility, and physical activity stage of change. An additional item asked who completed the questionnaire (the participant or a caregiver). This item was used as an inclusion criterion, and only surveys completed by the participants themselves were included in the analysis. The second section included an Arabic version of the Exercise Assessment and Screening for You (Ar-EASY) tool. The third section included two validated measures of physical activity: the International Physical Activity Questionnaire–Short Form (IPAQ-SF) and the Physical Activity Scale for the Elderly (PASE).

#### 2.2.1 Exercise assessment and screening for you

The EASY tool was developed by Resnick et al. (7) and was intended to support older adults by offering appropriate, safe, and efficient exercises based on their medical conditions. Additionally, the tool highlights the benefits of physical activity in this age group. It consists of six items, each with a yes or no response. Individuals with a score of one or higher are encouraged to engage in exercise. It provides individualized recommendations and safety advice specific to a given condition. If someone receives many “yes” responses, they are more likely to benefit from exercise and should develop their own exercise program. The original EASY tool was developed through expert panel consensus and demonstrated evidence of clinical and criterion validity, with scores showing significant associations with exercise participation and health-related outcomes in older adults. Notably, formal psychometric indices such as internal consistency or factor structure were not reported, reflecting its intended use as a screening and decision-support tool rather than a unidimensional measurement scale. Each item was scored as 0 (No) or 1 (Yes), and the total EASY score was calculated based on the sum of the validated items. Higher scores indicated greater exercise-related concerns.

#### 2.2.2 International Physical Activity Questionnaire (IPAQ) short form

The IPAQ was developed in the late 1990s as a globally applicable instrument for assessing physical activity levels among individuals aged 15–65 years across different settings (12, 13). The short form of the IPAQ comprises seven items that assess the time spent walking, moderate- and vigorous-intensity exercise, and sedentary activities over the past 7 days. The scoring system applies the Metabolic Equivalent of Task (MET); moderate activities raise the heart rate, respiratory rate, and sweating moderately for at least 10 min and have 3-6 METs, whereas vigorous activities result in vigorous increases in respiration rate, heart rate, and sweating for at least 10 min and are assigned more than six METs. Subsequently, MET-min per week were calculated for walking, moderate-intensity activity, and vigorous-intensity activity. We used the Arabic version of the IPAQ-SF, which has been validated and psychometrically tested to demonstrate valid and reliable measurement properties (13, 14).

A score was created by computing the total weekly MET minutes for vigorous, moderate, and walking activities using standard MET multipliers (8.0, 4.0, and 3.3, respectively). Values were truncated at 240 min per day per activity type, and durations under 10 min were recorded as zero.

#### 2.2.3 The Physical Activity Scale for the Elderly (PASE)

The Physical Activity Scale for the Elderly (PASE) is a tool specifically designed to assess physical activity levels in older adults. It consists of items that measure the frequency and duration of participation in leisure time, household, and occupational activities over the previous 7 days. The tool includes common activities in this age group, such as walking, recreational exercise, light and heavy household work, gardening, and caregiving. Each activity was weighted by its estimated energy expenditure, and the total PASE score was calculated by summing the weighted item scores, with higher scores indicating greater overall levels of physical activity (15). We used the Arabic version of the PASE, which has been validated and psychometrically tested, demonstrating valid and reliable metrics (16).

The PASE score was computed using a weighted formula as indicated in the official guidelines. The total PASE score reflected overall engagement in physical activity, with higher scores indicating greater activity levels.

### 2.3 Translation and cultural adaptation

After obtaining permission from the original developers, the translation and cultural adaptation of the EASY tool were conducted using a standardized forward–backward translation process (17). Two independent professional translators fluent in Arabic and English, one of whom had a medical background, translated the original English version into Arabic. The two forward translations were reviewed and harmonized through consensus meetings with S. M.A. and N. M.A. to produce a preliminary Arabic version. This preliminary version was then pilot-tested among 27 older adults to assess its clarity, comprehensibility, and cultural relevance, and minor linguistic refinements were made based on participant feedback.

The revised Arabic version was subsequently back-translated into English by an independent bilingual translator who was blinded to the original version. The back-translated version was reviewed by the original developers, who confirmed conceptual equivalence and raised no concerns regarding semantic or content accuracy. Following this iterative process, the final Arabic version of the EASY tool (Ar-EASY) was developed for psychometric evaluation (Supplementary material).

### 2.4 Psychometric assessment

The measurement properties evaluated in this study included structural validity, internal consistency, test–retest reliability, construct validity, known-groups validity, and measurement error. To assess test–retest reliability, the instrument was re-administered after an interval of approximately 2 weeks.

Construct validity was evaluated by testing a set of predefined hypotheses regarding the expected relationships between the EASY scale and established measures of physical activity. Therefore, the following hypotheses were formulated prior to analysis:

- H1: EASY scores will show a negative correlation with IPAQ scores.
- H2: EASY scores will show a negative correlation with PASE scores.
- H3: Higher EASY scores will be associated with lower odds of being physically active.
- H4: Individuals classified as physically inactive will have higher EASY scores than physically active individuals.

### 2.5 Statistical tests and software

All statistical analyses were conducted using RStudio (version 2024.9.1.394; Boston, MA, USA) with R version 4.4.2. Descriptive statistics were presented as medians and interquartile ranges (IQR) for continuous variables and frequencies (percentages) for categorical variables. The psychometric evaluation of the EASY scale included an exploratory factor analysis (EFA) using tetrachoric correlations to account for the items' binary nature. The minimum residual extraction method was applied. The number of factors was evaluated using parallel analysis. Additionally, confirmatory factor analysis to assess model fit, with model adequacy determined using χ^2^, CFI, TLI, RMSEA, and SRMR indices. Because the EASY items are dichotomous, the model was estimated using the weighted least squares mean and variance adjusted (WLSMV) estimator with theta parameterization, which is recommended for categorical indicators.

Internal consistency was assessed using ordinal alpha and omega coefficients. Test–retest reliability was evaluated using Cohen's kappa for item-level agreement and intraclass correlation coefficients for overall scale reliability. Internal consistency was further evaluated by calculating 95% confidence intervals for Cronbach's alpha and corrected item–total correlations for each EASY item. Measurement error was assessed by calculating the standard error of measurement (SEM) and the smallest detectable change at the individual level (SDC). Construct validity was examined through correlation with the IPAQ and PASE scores using the Spearman correlation test. Known-groups validity was examined by comparing EASY scores between physically active and physically inactive participants using the Wilcoxon rank-sum test, with effect sizes calculated to quantify the magnitude of group differences.

Logistic regression was used to identify predictors of physical activity status. The outcome variable was physical activity status (physically active vs. physically inactive) derived from the IPAQ classification. The predictor variables included the EASY score, age, gender, nationality, monthly income, and marital status. Rasch analyses were conducted using Winsteps software. The Rasch model assumes unidimensionality, local independence, and monotonicity of item responses; these assumptions were evaluated using fit statistics and residual-based dimensionality analyses. Missing data were minimal and handled using pairwise deletion for descriptive and correlation analyses. For confirmatory factor analysis, missing responses were handled using the WLSMV estimator implemented in the lavaan package, which treats all available data as present under a pairwise-presence approach. Statistical significance was set at *p* < 0.05.

## 3 Results

### 3.1 Demographic and functional characteristics of participants

**Figure 1:**
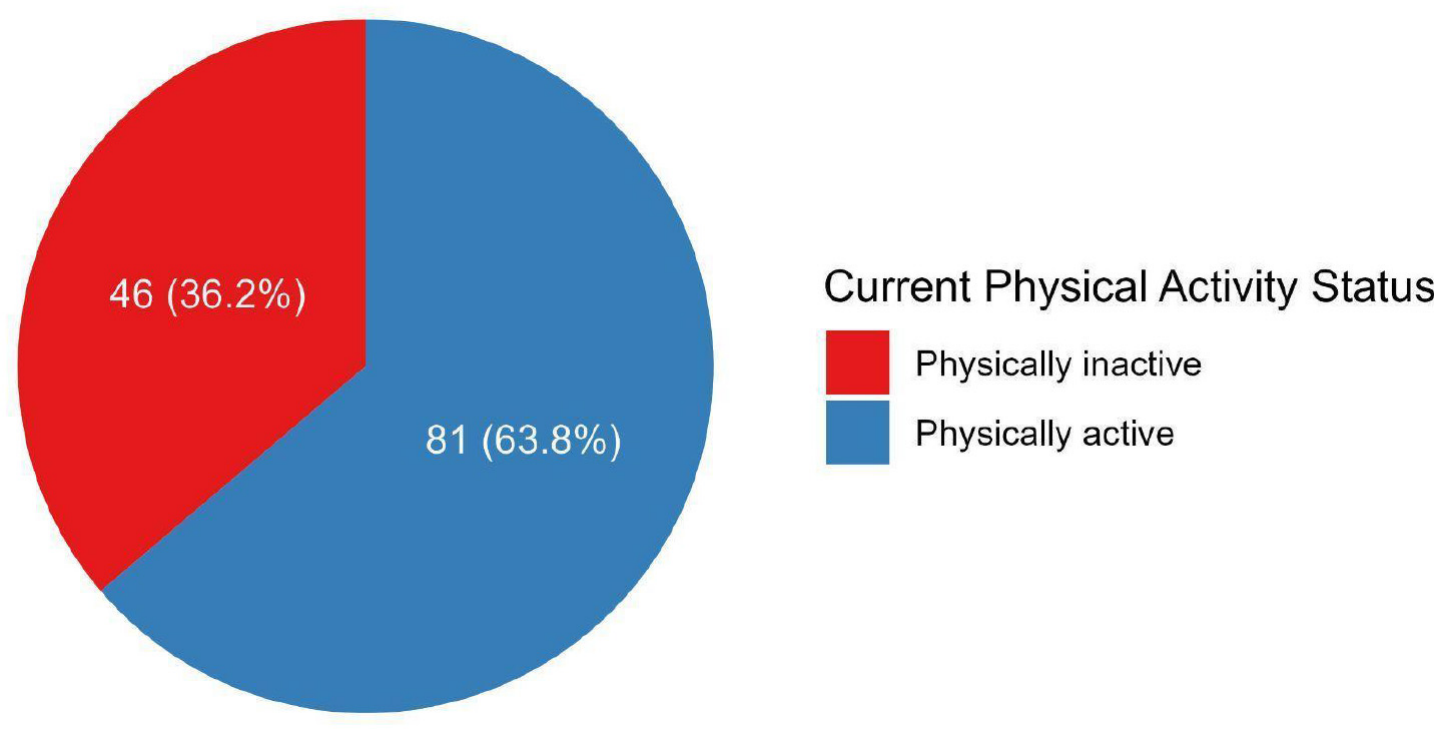
A pie chart depicting the frequencies and proportions of the categories of the current physical activity status.

We analyzed data from 127 older adults in the current study. The median age of participants was 60.0 years (IQR = 55.0 to 65.0). Of the participants, 58.3% were male and 41.7% were female. Most were Saudi nationals (89.8%) and married (89.0%). Regarding monthly income, 37.0% reported earnings between 5,000 and 14,999 SAR. Most participants (92.9%) were able to walk independently without assistance. Regarding the stages of physical activity behavior change, 48.8% reported being active for more than 6 months, whereas 15.0% had been active for less than 6 months, and 18.1% intended to start within the next 30 days (Table 1). Overall, the majority of participants (63.8%) were classified as physically active (*n* = 81), whereas 36.2% (*n* = 46) were classified as physically inactive based on their current physical activity status (Figure 1).

**Table 1:** Demographic and functional characteristics of older adults included in the Arabic EASY validation study at King Saud University Medical City, Riyadh, Saudi Arabia (*N* = 127).

Characteristic	Description
Age	60.0 (55.0–65.0)
Gender	
Male	74 (58.3%)
Female	53 (41.7%)
Nationality	
Saudi	114 (89.8%)
Non-Saudi	13 (10.2%)
Marital status	
Single	5 (3.9%)
Married	113 (89.0%)
Divorced	4 (3.1%)
Widowed	5 (3.9%)
Monthly income (SAR)	
<5,000	15 (11.8%)
5,000–14,999	47 (37.0%)
15,000–29,999	26 (20.5%)
30,000 or more	9 (7.1%)
Prefer not to disclose	30 (23.6%)
Functional status	
Walking independently without assistance	118 (92.9%)
Walking using a cane	6 (4.7%)
Partially using a wheelchair (can walk short distances)	2 (1.6%)
Others	1 (0.8%)
Physical activity stages of change	
Yes, for more than 6 months	62 (48.8%)
Yes, for less than 6 months	19 (15.0%)
No, but I intend to do it within the next 30 days	23 (18.1%)
No, but I intend to do it within the next 6 months	16 (12.6%)
No, I don't intend to do it within the next 6 months	7 (5.5%)

Median (Q1–Q3); *n* (%).

### 3.2 Rasch analysis

#### 3.2.1 Item fit and difficulty

Rasch analysis indicated a generally acceptable item fit. Infit mean square (MnSq) values ranged from 0.83 to 1.34, and Outfit MnSq values ranged from 0.73 to 1.65. Item difficulty estimates spanned from −1.89 to 1.69 logits, indicating a moderate range of item difficulty. Item Easy_3 was the easiest item (−1.89 logits), whereas Easy_5 was the most difficult (1.69 logits). Easy_3 demonstrated a borderline misfit (Infit = 1.34; Outfit = 1.65), whereas all other items showed a satisfactory fit to the Rasch model (Table 2).

**Table 2:** Summary of Rasch model fit and reliability indices.

Item	Item measure (logits)	SE	Infit MnSq	Outfit MnSq	PTMEA Corr
EASY_3	−1.89	0.23	1.34	1.65	0.40
EASY_1	−0.40	0.25	0.95	0.89	0.55
EASY_4	−0.21	0.26	0.83	0.76	0.60
EASY_2	0.07	0.27	0.92	0.85	0.53
EASY_6	0.74	0.32	0.96	0.77	0.47
EASY_5	1.69	0.43	0.97	0.73	0.39

#### 3.2.2 Rasch reliability and separation

The item reliability was 0.92, with an item separation index of 3.46, indicating good item hierarchy and stability. Person reliability and person separation were both 0.00, indicating a limited ability of the scale to distinguish respondents across different levels of the latent trait in this sample.

#### 3.2.3 Dimensionality

Principal component analysis (PCA) of the Rasch residuals showed that the Rasch dimension explained 27.6% of total variance. The first residual contrast had an eigenvalue of 1.52, suggesting borderline evidence of unidimensionality. Easy_3 exhibited the highest standardized residual loading (0.96), indicating potential secondary dimensionality.

#### 3.2.4 Item difficulty hierarchy and targeting

The hierarchy of item difficulty indicated that Easy_3, Easy_1, and Easy_4 were relatively easy to endorse, whereas Easy_6 and Easy_5 were more difficult. Person–item maps suggested that the items were reasonably targeted to the distribution of respondent abilities.

### 3.3 Exploratory factor analysis

Exploratory factor analysis (EFA) was first conducted on the six-item EASY scale using tetrachoric correlations to account for the dichotomous nature of the items. Factor extraction was performed using the minimum residual method. Parallel analysis suggested a two-factor solution; however, inspection of factor loadings indicated that five items (Easy_1, Easy_2, Easy_4, Easy_5, and Easy_6) loaded moderately to strongly on a single factor (loadings 0.52–0.74), while Easy_3 showed a weak negative loading (−0.16) and minimal communality (h^2^ = 0.03). These findings suggested that Easy_3 did not adequately represent the underlying construct measured by the scale. After removing Easy_3, EFA was repeated on the remaining five items. The analysis supported a unidimensional structure, with factor loadings ranging from 0.50 to 0.77 and the single factor explaining 40.6% of the variance.

### 3.4 Confirmatory factor analysis

During the initial one-factor CFA, which included all six EASY items, one item demonstrated poor psychometric performance. Specifically, an item asking about whether a participant had ever been told that they had high blood pressure (Easy_3) showed a negative and very weak standardized factor loading (Std.all = −0.17, *p* = 0.39), indicating an inverse and non-meaningful relationship with the underlying construct. In addition, this item exhibited an extremely high residual variance (Std = 0.97). Reliability indices for the full six-item model were also suboptimal (ordinal α = 0.64; ω = 0.43; AVE = 0.39). Therefore, this item was removed from subsequent analyses to improve the factorial validity and internal consistency of the EASY scale (Table 3).

**Table 3:** Confirmatory factor analysis results and reliability indices for the five-item Arabic EASY scale.

Component	Statistic	Value
Model fit	χ^2^ (df = 5), *p*-value	3.83, *p* = 0.57
	CFI	1
	TLI	1.08
	RMSEA (90% CI)	0.00 (0.00–0.11)
	SRMR	0.076
Standardized factor loadings	Easy_1	0.53
	Easy_2	0.65
	Easy_4	0.75
	Easy_5	0.71
	Easy_6	0.59
Reliability and validity	Ordinal alpha	0.77
	Omega	0.57
	Average variance extracted (AVE)	0.44

Subsequently, the new model demonstrated excellent fit to the data, with a non-significant chi-square test and optimal incremental and absolute fit indices (CFI = 1.00, TLI = 1.08, RMSEA = 0.00, and SRMR = 0.076). The TLI value, which slightly exceeds 1.0, likely reflects small-sample estimation effects and the simplicity of the one-factor model rather than true overfitting. All items showed moderate to strong standardized factor loadings (range: 0.53–0.75). The item thresholds were statistically significant. Reliability analysis indicated acceptable internal consistency for categorical data, with an ordinal alpha of 0.77, while composite reliability estimates (omega ≈ 0.57) and average variance extracted (AVE = 0.44) were modest (Table 2).

### 3.5 Scores of study instruments

The EASY score at baseline had a median of 0.0 (IQR = 0.0–1.0), with a mean of 0.8 (SD = 1.1), and ranged from 0 to 4. At follow-up (*n* = 25), the EASY score had a median of 1.0 (IQR = 0.0–1.0). The IPAQ score (*n* = 49) had a median of 1,422.0 MET-min/week (IQR = 720.0–3,519.0), and the PASE score (*n* = 60) had a median of 105.3 (IQR = 59.2–147.4) (Table 4).

**Table 4:** Description of EASY, IPAQ, and PASE scores among participants at King Saud University Medical City, Riyadh, Saudi Arabia.

Characteristic	Description
Easy score, *n* = 124	
Median (Q1–Q3)	0.0 (0.0–1.0)
Mean (SD)	0.8 (1.1)
Min–Max	0.0–4.0
Easy score (time 2), *n* = 25	
Median (Q1–Q3)	1.0 (0.0–1.0)
Mean (SD)	0.9 (1.0)
Min–Max	0.0–3.0
IPAQ score, *n* = 49	
Median (Q1–Q3)	1,422.0 (720.0–3,519.0)
Mean (SD)	2,460.6 (2,527.8)
Min–Max	0.0–11,128.8
PASE score, *n* = 60	
Median (Q1–Q3)	105.3 (59.2–147.4)
Mean (SD)	106.4 (62.1)
Min–Max	5.0–289.2

IPAQ, International Physical Activity Questionnaire; PASE, Physical Activity Scale for the Elderly; SD, standard deviation.

### 3.6 Construct validity (correlation between the EASY score and IPAQ and PASE scores)

**Figure 2:**
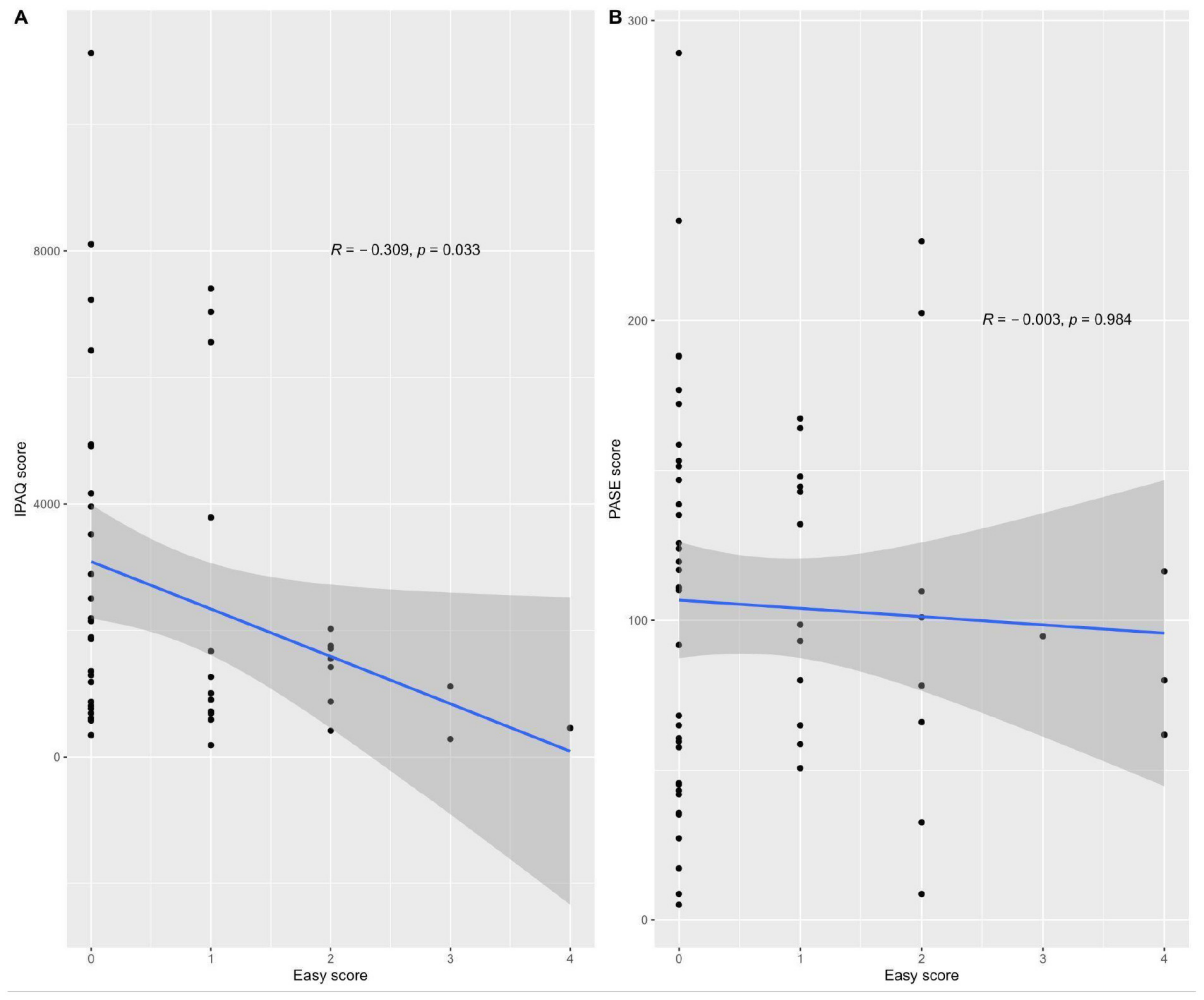
Scatterplots depicting bivariate correlations between EASY and IPAQ scores **(A)** and EASY and PASE scores **(B)**.

As shown in Figure 2, there was a statistically significant negative correlation between EASY scores and IPAQ scores (*r* = −0.309, *p* = 0.033; Figure 2A), indicating that higher EASY scores were associated with lower levels of self-reported physical activity. In contrast, no significant correlation was found between EASY scores and PASE scores (*r* = −0.003, *p* = 0.984; Figure 2B).

### 3.7 Known-groups validity

Known-groups validity was assessed by comparing EASY scores between participants classified as physically inactive and physically active. Physically inactive participants demonstrated significantly higher EASY scores than physically active participants [median 1.0 [IQR = 0.0 to 2.0] vs. 0.0 [IQR = 0.0 to 1.0], *p* < 0.001]. The effect size was moderate (r = 0.33), indicating that the EASY scale was able to discriminate between groups with expected differences in exercise readiness.

### 3.8 Predictors of being physically active

The EASY score was inversely associated with being physically active, and the association was significant after adjustment for demographic variables (OR = 0.53, 95% CI, 0.34–0.78, *p* = 0.002) (Table 5).

**Table 5:** Logistic regression analysis of predictors of physical activity status among older adults.

Characteristic	OR	95% CI	*p*-value
Easy score, *n* = 124	0.53	0.34, 0.78	0.002
Age	1.02	0.96, 1.08	0.589
Gender			
Male	Reference	Reference	
Female	2.11	0.79, 6.04	0.148
Nationality			
Saudi	Reference	Reference	
Non-Saudi	0.60	0.17, 2.12	0.416
Monthly income (SAR)			
<5,000	Reference	Reference	
5,000–14,999	1.27	0.30, 5.20	0.738
15,000–29,999	0.98	0.19, 4.75	0.975
30,000 or more	1.44	0.19, 14.0	0.731
Prefer not to disclose	0.79	0.17, 3.37	0.753
Marital status			
Single/divorced/widowed	Reference	Reference	
Married	0.74	0.16, 2.92	0.680

CI, Confidence Interval; OR, Odds Ratio.

### 3.9 Test–retest reliability of the EASY scale

At the item level, substantial agreement was observed for Easy_1 (κ = 0.60, *p* = 0.003) and Easy_5 (κ = 0.65, *p* < 0.001). Fair agreement was observed for Easy_2 (κ = 0.36, *p* = 0.053), Easy_4 (κ = 0.32, *p* = 0.116), and Easy_6 (κ = 0.36, *p* = 0.053) (Table 6). At the scale level, the test–retest reliability was moderate, with an intraclass correlation coefficient of 0.58 (95% CI, 0.25 to 0.80, *p* = 0.001). Measurement error analysis showed an SEM of 0.69 and an SDC at the individual level of 1.91 points.

**Table 6:** Item-level test–retest reliability of the EASY scale (Cohen's kappa) (*n* = 25).

Item	Cohen's κ	z-value	*p*-value	Agreement level
Easy_1	0.60	3.00	0.003	Substantial
Easy_2	0.36	1.94	0.053	Fair
Easy_4	0.32	1.57	0.116	Fair
Easy_5	0.65	3.39	<0.001	Substantial
Easy_6	0.36	1.94	0.053	Fair

Cohen's κ values were interpreted as follows: <0.40 = poor to fair agreement, 0.41–0.60 = moderate agreement, 0.61–0.80 = substantial agreement, and >0.80 = excellent agreement.

Internal consistency of the EASY scale was assessed using Cronbach's alpha. The five-item version of the scale demonstrated moderate internal consistency (Cronbach's α = 0.55, 95% CI 0.41–0.66). Corrected item–total correlations ranged from 0.28 to 0.36, indicating acceptable item discrimination. None of the items substantially improved internal consistency when removed, as Cronbach's alpha ranged from 0.46 to 0.52 when individual items were deleted.

### 3.10 Discriminative ability of the EASY score

**Figure 3:**
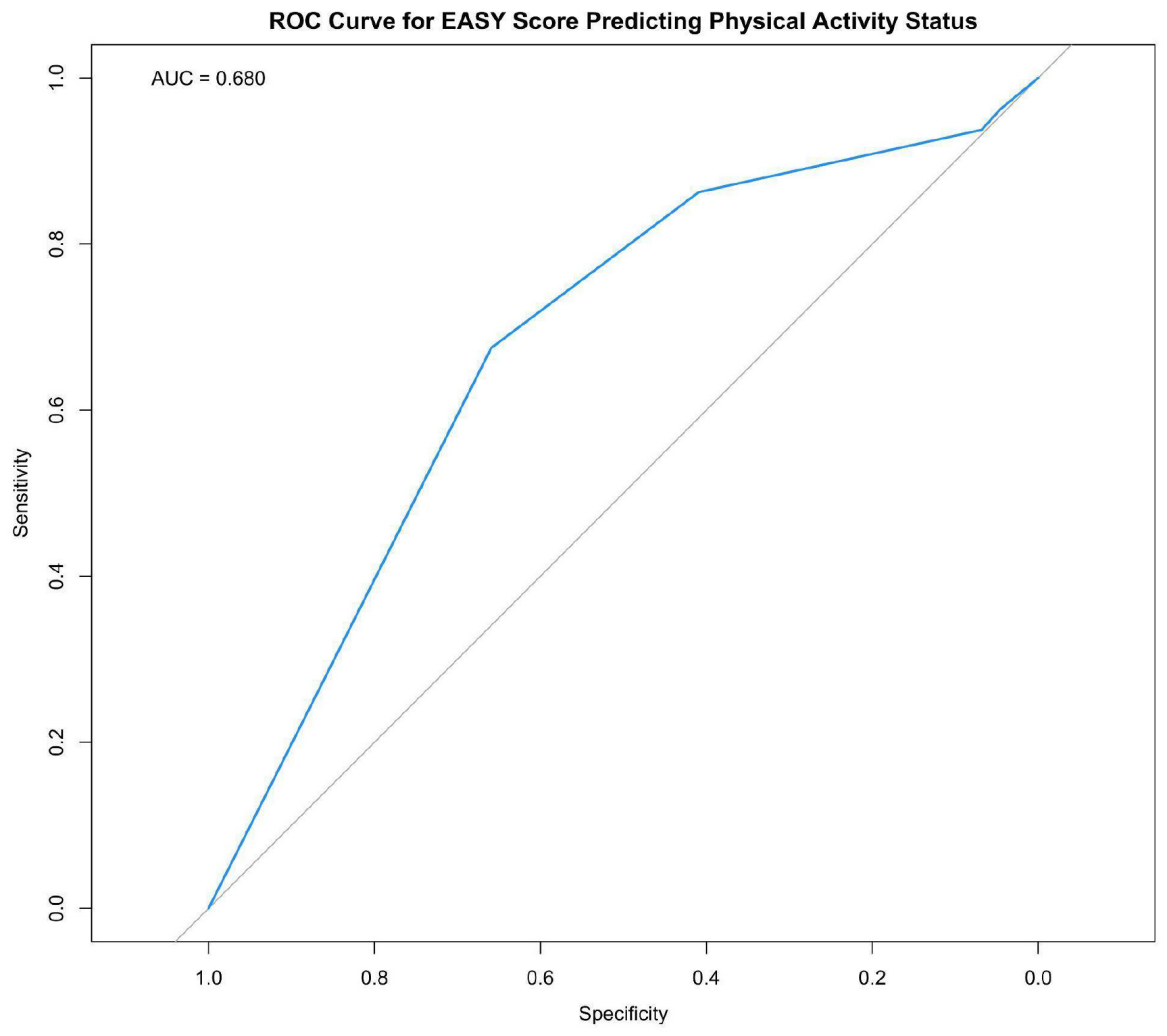
Receiver operating characteristic curve of the Arabic EASY score for discriminating physical activity status. AUC, Area Under the Curve.

As illustrated in Figure 3, the receiver operating characteristic (ROC) curve shows acceptable separation from the diagonal reference line. The area under the curve (AUC) was 0.680, indicating fair discriminative ability. The 95% confidence interval (CI) for the AUC ranged from 0.587 to 0.773.

## 4 Discussion

The Exercise Assessment and Screening For You (EASY) tool utilizes six screening questions to signpost older adults toward tailored physical activity regimens with consideration of existing health conditions and disabilities. This study aimed to translate, culturally adapt, and evaluate the psychometric properties of the Arabic version of the EASY (Ar-EASY) tool, with findings supporting its potential as a brief, clinically applicable screening instrument rather than a comprehensive psychometric scale. To our knowledge, this is the first study to translate and validate the EASY tool in an Arabic-speaking population. Overall, the results demonstrate acceptable structural validity and preliminary clinical utility, although some measurement properties were modest.

Rasch analysis showed overall good item fit (Infit MnSq range 0.83–1.34 and Outfit MnSq range 0.73–1.65) with borderline unidimensionality (27.6% explained variance and first contrast eigenvalue of 1.52). However, person reliability was notably low, indicating a limited ability of the scale to discriminate between individuals in this sample, likely due to restricted variability and the relatively homogeneous clinical population, and to differentiate between individuals across levels of exercise readiness. This limitation should be considered when interpreting the tool's ability to distinguish between individuals in similar clinical populations. We noted a degree of multidimensionality likely introduced by one item, Easy_3, which had the highest standardized residual loading (0.96). We performed CFA to better understand the underlying structure of items and to explain potential secondary dimensionality. The CFA revealed that Easy_3 showed a weak negative factor loading on the underlying construct, with a high residual variance. This finding suggests that Easy_3 may not align conceptually with the latent construct measured by the remaining items, which appear to reflect perceived symptoms and functional concerns related to exercise. Although the first contrast eigenvalue was below the commonly accepted threshold of 2.0, the relatively low explained variance suggests that the scale may not be strongly unidimensional. Therefore, the possibility of multidimensionality should be considered when interpreting the construct measured by the Ar-EASY.

Easy_3 assesses whether the participant had ever been told that they had high blood pressure; therefore, this finding may also occur in the English-language model, as Easy_3 is conceptually distinct from the other items. It is the only item that assesses an external objective measure (whether the participant has been told about high blood pressure), whereas the other items each contain a component of the participants' self-awareness of their symptoms or concerns. It is also the only item that has a retrospective component, as it asks about prior diagnosis of high blood pressure, meaning that it may not solely reflect the participant's current status at the time of response.

Easy_3 was also the most frequently endorsed item (−1.89 logits in Rasch analysis), resulting in reduced variability for CFA. This finding is not unexpected, given the high prevalence of hypertension among older adults in Saudi Arabia (18). Similar challenges with clinically oriented items have been reported in validation studies of screening tools, where diagnosis-based items may not align well with latent constructs reflecting perceived functional status or behavioral readiness. Notably, our study recruited participants from a limited pool that included patients being followed up in family medicine and specialized geriatric clinics, rather than from the general population, which may have biased the sample toward a higher proportion of affirmative responses to Easy_3. In contrast, the original study describing the EASY tool recruited participants from a continuing care retirement community equipped with age-appropriate fitness facilities, including a fitness room, pool, and trainers likely leading to a different type of demographic limitation compared with our study (7). The narrow sampling pool may also explain why person separation reliability remained low during Rasch analysis, despite the removal of Easy_3 (19, 20).

While the removal of Easy_3 improved statistical model fit, it may reduce the conceptual completeness of the instrument and limit comparability with the original version. This trade-off between psychometric optimization and conceptual coverage should be considered when interpreting the findings.

The removal of Easy_3, leaving only five items, produced an improved model fit (CFI = 1.00), demonstrating the validity of the model. Although the model demonstrated excellent fit indices, these should be interpreted cautiously given the small sample size and limited number of items, which may artificially inflate model fit statistics (21). Similarly, the AVE and measures of reliability improved with the modified model, although they remained modest to moderate. This led to the decision to format the translated EASY tool as a five-item questionnaire with a caveated appendix for the sixth item for reference. Internal consistency was modest, which may be expected given the limited number of items and dichotomous response structure (22). These findings are consistent with recent validation studies of brief screening tools, which often report modest internal consistency and discriminative ability due to their screening-oriented design.

Construct validity was assessed by comparing the EASY score with the IPAQ and PASE scores. The negative correlation with IPAQ scores and the lack of a significant correlation with PASE may appear inconsistent; however, this is likely explained by conceptual differences between the instruments, given that these tools assess different constructs. While the EASY tool was created to assess suitability for exercise and guide participants toward safe forms of physical activity, the IPAQ and PASE tools aim to directly assess participants' engagement in physical activity. The choice to use these tools for comparison was necessitated by a lack of validated Arabic-language instruments designed to perform a function similar to that of the EASY tool. The inverse association between the EASY score and physical activity may be explained by the fact that the EASY questions relate to concepts that may be perceived as barriers to engaging in physical activity, meaning that a high EASY score is likely to be a proxy measure of hesitancy to engage in physical activity. If this explanation is correct, the EASY tool may also serve as a screening instrument to identify individuals at risk of inactivity. An alternative explanation is that engaging in physical activity may lead to improvements in patient-reported symptoms (items 1, 2, 4, and 5) and reduce concerns about initiating an exercise program (item 6). These findings are consistent with prior evidence showing that screening tools assessing perceived readiness may demonstrate only modest correlations with behavioral physical activity measures (23), and should be interpreted in light of WHO physical activity frameworks (24, 25).

The AUC of 0.68 indicates modest discriminative ability. While this level of performance is not strong, it is consistent with the intended use of the EASY as a screening tool rather than a diagnostic instrument (19, 22).

The strengths of our study include analysis with both Rasch and CFA, which are additional analytical approaches compared with those used in the original English language tool and allow for improved understanding of the individual items. The limitations of our study include a relatively small sample size from a limited recruitment pool, which may not fully represent the Arabic-speaking population. Although the rule of 10–20 participants per item is adequate for EFA, confirmatory factor analysis generally requires larger samples to ensure stable parameter estimation. Furthermore, formal content validity indices (e.g., CVI, CVR) were not calculated, which may limit the strength of evidence for content validity. The study also did not assess longitudinal validity beyond test-retest reliability, which should be considered in future research. In terms of construct validity testing, we were limited by a lack of known Arabic-language tools that assess analogous concepts, leading to the selection of instruments that were weakly correlated with the EASY.

Despite these limitations, it is important to emphasize that the EASY tool is intended as a brief screening and guidance instrument rather than a unidimensional scale measuring a single latent construct. Therefore, modest internal consistency and discriminative ability should be interpreted within the context of its intended clinical use. The Arabic version of the EASY tool provides a feasible, culturally adapted, and clinically applicable screening approach to support safe physical activity recommendations among older adults. Future research should focus on evaluating its performance in larger and more diverse populations, as well as exploring its responsiveness and longitudinal validity. Overall, the psychometric findings of the present study suggest mixed evidence of validity. While structural analyses supported a unidimensional five-item model, other indicators were less robust. Person reliability was extremely low, indicating limited ability to distinguish between individuals. Construct validity findings were inconsistent, with a moderate association observed with IPAQ but no correlation with PASE. Test–retest reliability was also moderate.

## 5 Conclusion

The Arabic version of the EASY scale demonstrated mixed but generally acceptable psychometric properties as a brief screening tool, with limitations in reliability and construct validity. The five-item EASY tool may serve as a brief screening instrument to guide physical activity recommendations for older Arabic-speaking adults, although further validation in larger and more diverse populations is warranted.

## Data Availability

The raw data supporting the conclusions of this article will be made available by the authors, without undue reservation.
