# Supplementary material for: Arabic exercise assessment and screening for you tool: development, validation, and cultural adaptation among older adults in Saudi Arabia
Source: Front Med (Lausanne). 2026 Apr 13;13:1815705. doi: 10.3389/fmed.2026.1815705 (PMC13111066; doi:10.3389/fmed.2026.1815705)
Supplement: Supplementary file 1 [file Supplementary_file_1.pdf]

تقييم التمارين والفحص من أجلك

الأسئلة	نعم	لا
**1- هل تشعر بآلام أو ضيق أو ضغط في الصدر أثناء القيام بنشاط جسدي (المشي، صعود السلالم، الأعمال المنزلية، الأنشطة المشابهة)؟**	( )	( )
**2- في الوقت الحاضر هل تصاب بدوخة أو دوار؟**	( )	( )
**3- هل سبق وقيل لك أنك تعاني من ارتفاع ضغط الدم؟**	( )	( )
**4- هل لديك ألم أو تيبّس أو تورّم يحد من حركتك أو يمنعك من القيام بما تريد أو تحتاج فعله؟**	( )	( )
**5- أثناء الوقوف أو المشي هل تتعرض للسقوط أو تشعر بعدم اتزان أو تستخدم أجهزة مساعدة (كالعكاز أو الكرسي المتحرك)؟**	( )	( )
**6- هل يوجد سبب صحي لم يُذكر يجعلك تقلق بشأن البدء في برنامج تمارين رياضية؟**	( )	( )

إذا أجبت بـ "لا" عن جميع أسئلة هذه الاستبانة، فاتبع **الخطوات الثلاثة** الآتية لبدء أو متابعة برنامجك الرياضي:

١. اختر أنشطة التي تتناسب مع روتينك اليومي.
٢. ليكن هدفك أن تكون نشطًا لمدة ٣٠ دقيقة يوميًا في أغلب أيام الأسبوع (من الأفضل العمل على الوصول إلى هذا الهدف ببطء).
٣. راجع إرشادات السلامة في هذه الباقة.

تُقدم هذه الأداة لكبار السن والمختصين توصيات بأنشطة مناسبة وآمنة بناءً على عوامل الخطورة المعطاة. الأداة تتبع نهجًا متكاملًا في اختيار برنامج التمرين الرياضي المناسب لكل شخص، وتعيين عوامل الخطورة المحتملة المرتبطة بممارسة الرياضة، وتقديم إرشادات للسلامة، والتشجيع على التواصل مع مقدمي الرعاية الصحية.

بوجه عام، ليس من الضروري زيارة مقدم للرعاية الصحية قبل بدء ممارسة أنشطة بدنية يومية خفيفة أو متوسطة الشدة، إلا أننا نوصيك بالتحدث مع مقدم الرعاية الصحية الخاص بك حول حالتك الصحية والرياضة التي تمارسها ضمن زياراتك المنتظمة له.

إذا كنت قد أجبت بـ "نعم" عن أي سؤال من الأسئلة البسيطة، اتبع ورقة التوصيات لكي تمارس الرياضة بأمان بناءً على حالتك المَرضيّة. من الأفضل دائمًا قراءة إرشادات السلامة ومعرفة التمارين الرياضية التي يرى الخبراء أنها الأنسب بناءً على الأمراض المُعيّنة. مع كل سؤال، أضفنا رابطًا للحصول على المزيد من المعلومات. تحدث مع مقدم الرعاية الصحية الخاص بك عن برنامج التمرين الرياضي الذي تتبعه أثناء زياراتك المنتظمة له.

جامعة الملك سعود
King Saud University

الأسئلة	نعم
**١- هل تشعر بآلام أو ضيق أو ضغط في الصدر أثناء القيام بنشاط جسدي (المشي، صعود السلالم، الأعمال المنزلية، الأنشطة المشابهة)؟**	إذا أجبت بـ "نعم" عن هذا السؤال وكانت هذه مشكلة جديدة، راجع مقدم الرعاية الصحية الخاص بك أولًا قبل بدء ممارسة أي تمارين رياضية. اسأل مقدم الرعاية الصحية الخاص بك: "هل توجد تمارين رياضية يجب أن لا أمارسها"؟ اعمل مع طبيبك لتحديد الأنشطة المناسبة لك. إذا لم تكن المشكلة الصحية جديدة أو إذا تم تقييمها في السابق، يمكنك أن تبدأ أو تستمر في ممارسة برنامجك الرياضي.
**٢- في الوقت الحاضر هل تصاب بدوخة أو دوار؟**	إذا أجبت بـ "نعم"، نوصي بأن تتحدث مع مقدم الرعاية الصحية الخاص بك قبل بدء برنامج نشاط بدني جديد. اسأل ما إذا كانت هناك تمارين يجب أن لا تمارسها. اعمل مع طبيبك في تعيين التمارين الرياضية المفيدة لك.
**٣- هل سبق وقيل لك أنك تعاني من ارتفاع ضغط الدم؟**	إذا لم يُعايَن ضغط دمك من قبل ممارس صحي خلال الستة أشهر الماضية، فتأكد من زيارة مقدم للرعاية الصحية لقياسه. إذا أجبت بـ "نعم"، يمكنك أن تستمر في ممارسة الرياضة لتحسين صحة قلبك عامة والوقاية من الأمراض.
**٤- هل لديك ألم أو تيبّس أو تورّم يحد من حركتك أو يمنعك من القيام بما تريد أو تحتاج فعله؟**	إذا أجبت بـ "نعم"، فاستمر في ممارسة تمارينك الرياضية للوقاية من تفاقم التهاب المفاصل لديك والمساعدة في إدارة الألم. وإذا كنت مصابًا بهشاشة العظام، فتجنب تمارين التمدد التي تقتضي ثني العمود الفقري أو الانثناء من الوسط، وتجنب الحركات النّفضِيّة السريعة.
**٥- أثناء الوقوف أو المشي هل تتعرض للسقوط أو تشعر بعدم اتزان أو تستخدم أجهزة مساعدة (كالعكاز أو الكرسي المتحرك)؟**	إذا أجبت بـ "نعم"، نوصي بأن تتحدث مع مقدم الرعاية الصحية الخاص بك قبل بدء برنامج نشاط بدني جديد. اسأل ما إذا كانت هناك تمارين يجب أن لا تمارسها. اعمل مع طبيبك في تعيين تمارين رياضية مفيدة لك.
**٦- هل يوجد سبب صحي لم يُذكر يجعلك تقلق بشأن البدء في برنامج تمارين رياضية؟**	إذا أجبت بـ "نعم"، شارك هذه المعلومات مع مقدم الرعاية الصحية الخاص بك. أغلب الأسباب المانعة لممارسة الرياضة يمكن التعامل معها لتتمكن من بدء برامج رياضي يعزز صحتك ورفاهيتك بصفة عامة.

Do not use without permission. Please ensure proper citation of both the original paper by Resnick *et al.* (2008) and the Arabic validation paper by Alsaad *et al.* (2026) when using this version in your article.
